# Leaf mechanical properties as potential predictors of leaf-litter decomposability

**DOI:** 10.48130/FR-2023-0021

**Published:** 2023-09-20

**Authors:** Wenshan Li, Zhenya Liu, Jianfeng Zhao, Liangfan Ma, Jiahao Wu, Jinfeng Qi, Hang Wang

**Affiliations:** 1 Yunnan Key Laboratory of Plateau Wetland Conservation, Restoration and Ecological Services, Southwest Forestry University, Kunming 650224, People's Republic of China; 2 Forestry College, Fujian Agriculture and Forestry University, Fujian 350002, People's Republic of China; 3 Department of Economic Plants and Biotechnology, Yunnan Key Laboratory for Wild Plant Resources, Kunming Institute of Botany, Chinese Academy of Sciences, Kunming 650201, People's Republic of China; 4 Key Lab of Urban Environment and Health, Institute of Urban Environment, Chinese Academy of Sciences, Xiamen 361021, People's Republic of China

**Keywords:** Litter decomposition, Leaf functional trait, Litter quality, Litter physical strength trait, Leaf toughness

## Abstract

The mechanical resistance of plant leaves to herbivores and physical disturbances have a lasting legacy impact on leaf-litter decomposition rates and nutrient leaching. However, in the past, leaf mechanics were seldom considered as key factors in regulating ecological processes related to leaf-litter decomposition. In this paper, we explored the physical strength traits of leaves, which are essential components of plant functional traits. These traits are primarily manifested through three mechanical properties: force to punch, force to tear, and work to shear. We discuss their potential applications in order to better understand trait-based factors influencing leaf-litter decomposition as well as other ecological processes. Their ecological connections and distinctions from other widely discussed plant functional traits relevant to decomposition processes were also addressed. By conducting an extensive literature survey, we further showed the importance and irreplaceability of leaf physical strength traits as potential predictors of leaf-litter decomposability compared with commonly measured plant chemical traits (e.g., carbon, nitrogen, and lignin). Recognizing leaf mechanics as vital yet previously overlooked determinants of ecological processes governing leaf-litter decomposition, we propose incorporating this set of traits into existing predictive models to improve the explanatory capability of plant species traits in regulating leaf-litter decomposition processes.

As one of the two major carbon-transforming processes on Earth, plant decomposition remains a continuously evolving field in forestry research^[1]^. Its trajectory can be predicted through the well-established triangular model, which integrates litter quality, climatic conditions, and decomposers as the three pivotal factors regulating the rates and patterns of decay^[2]^. Extensive research has been conducted on these factors, both individually and in terms of their interactions. Of note, global metadata encompassing 818 species from 66 decomposition experiments conducted across six continents have unequivocally demonstrated that species-driven differences in litter mass loss outweigh climate-driven variations^[3]^. This highlights the paramount significance of interspecific variations in plant functional traits as inherent drivers of the complex and dynamic processes underlying litter decomposition.

Plant functional traits, including a set of matrices and measures, reflect plant ecological strategies in adapting to local environments. The growth of large, flat leaves is primarily driven by the need for light interception and carbon assimilation^[4]^. Such leaf structures, however, are susceptible to damage from herbivores and physical disturbances such as wind-induced forces^[4]^. Leaf mechanical resistance thus serves as a crucial ecological strategy, or more precisely, a defense strategy against potential risks of injury^[5]^. Leaf mechanical resistance to herbivores and physical disturbances affects the decomposition processes of leaf litter, given the widely recognized fact that leaves with high physical strength tend to exhibit poor litter quality, resulting in high resistance to decay^[5]^. This phenomenon primarily occurs through the legacy effects of living plant traits once these leaves enter the detritus trophic pathway, which is mainly driven by decomposers that colonize leaf litter^[6]^. As such, ecological processes related to leaf-litter decomposition in different species exhibit a close connection with their interspecific variations in leaf mechanical resistance.

Surprisingly, most studies have predominantly focused on leaf chemical traits (such as carbon, nitrogen, phosphorus, lignin, carbon-to-lignin ratio, and tannin) as candidate predictors of leaf-litter decomposability, whereas leaf physical strength traits have been largely overlooked or rarely examined in the field. We conducted an extensive literature survey through the Web of Science database (http://apps.webofknowledge.com/) to identify pertinent studies released between 2010 and 2022. The search terms were 'decomposition' AND 'litter', which resulted in a total of 2,246 papers. These publications were screened by synthesizing those that assessed plant traits as predictors of litter decomposability. To meet this criterion, the study had to include both the litter decomposition rate (or metrics such as litter mass remaining or mass loss) and plant functional traits, and the relationships between them had to be examined within the context of a litter decomposition experiment. After screening, 170 publications were compiled, which are detailed in Supplemental Table S1. Among them, we only identified 23 papers that investigated the physical strength traits of leaves and their correlations with decay rates and patterns (Fig. 1). Upon further investigation of these studies, we found that nearly all of them demonstrate the critical role of leaf physical strength traits in explaining the decomposition rates or dynamic patterns of leaf-litter samples. For instance, one study examined the leaf litter of 203 tree species in a subtropical forest and showed that the relative importance of physical traits in affecting litter decomposition was equivalent to or even higher than that of nutrient-related traits at the very early and late decomposition stages^[7]^. Moreover, by examining the relationships among leaf functional traits and mass loss during the early phases of leaf-litter decomposition in 12 woody plant species^[8]^, it was shown that physical traits predicted litter mass loss as well or better than chemical traits. In this study, the term 'leaf toughness' is adopted and frequently used in several studies^[9,10]^ to refer to the punching force to penetrate a leaf. In other studies^[11,12]^, the same term has been used, but as an indicator of leaf tensile strength. Our literature surveys revealed that different physical properties of leaves have been measured to indicate 'leaf toughness', making it difficult to compare across these studies. Moreover, even the term 'leaf toughness' is imprecise when used to describe the leaf mechanical properties of plants through the act of punching holes in leaves. According to Wright & Illius^[13]^, toughness refers to the resistance of a material to the propagation of a crack. It is measured by the energy required to propagate a crack through a material sample, expressed in joules (J). By definition, toughness involves dividing the work to fracture by the cross-sectional area of the cracked surface. This calculation yields the energy per unit area of fracture (J·m^−2^), commonly referred to as the specific work of fracture or the material's toughness^[14]^.

**Figure 1:**
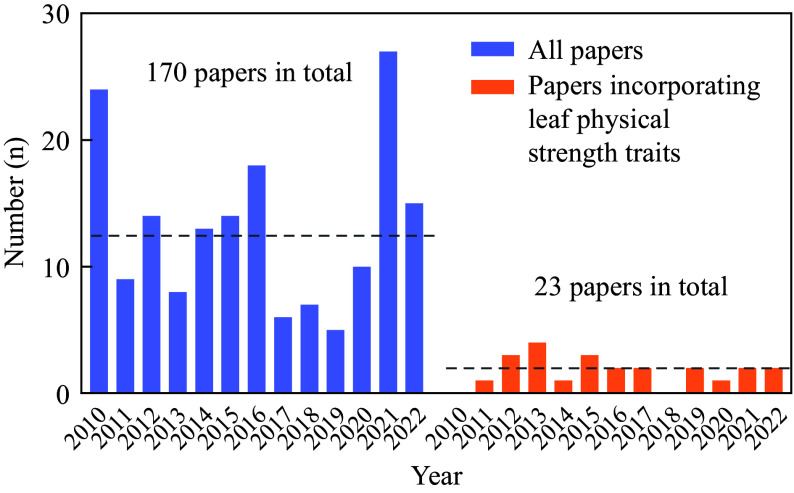
Literature survey of papers studying leaf-litter decomposition in association with plant functional traits in fresh leaves as predictors of litter decomposability from 2010−2022. Those papers incorporating leaf physical strength traits (orange bars on the right side) are compared with all papers (blue bars on the left side) in the field.

According to the latest handbook for plant trait measurement^[15]^, leaf physical strength traits can be quantified by evaluating three commonly measured mechanical properties: force to punch (*F*_*p*_, in N·m^−1^), force to tear (*F*_*t*_, N·m^−1^), and work to shear (*W*_*s*_, J·m^−1^). *F*_*p*_ is the force needed to force a punch through a leaf, *F_t_* is the force needed to tear a leaf, and *W*_*s*_ reflects the force needed to cut a leaf at a constant angle and speed. The calculation is based on the equations according to Onoda et al.^[5]^, as follows:

Force to punch (*F*_*p*_, in N·m^−1^) = Force / circumference of punch

Force to tear (*F*_*t*_, N·m^−1^) = Force / fracture width

Work to shear (*W*_*s*_, J·m^−1^) = Work / fracture length

The 'Force' can be readily obtained from the equipment. To determine the 'Work' in units of J, the integral area under the curve (force × displacement) should be calculated. Detailed descriptions outlining the determination of these mechanical properties can be found in this referenced handbook. Inspired by this, we recommend categorizing all of these mechanical properties in terms of 'leaf physical strength traits', which may represent a more precise terminology than 'leaf toughness'.

For clarity, we illustrated the common tests for these mechanical properties upon fully mature fresh leaf samples (see Fig. 2). For each test, we specifically identified the locations on the leaf surfaces where these tests are recommended to be conducted to maintain consistency across different samples in leaf-litter decomposition experiments. A test was conducted on leaves from *Eucalyptus globulus*, yielding average values of 375 N·m^−1^ for *F*_*p*_ and 945 N·m^−1^ for *F*_*t*_. For *W*_*s*_, it was 0.979 J·m^−1^ when excluding the midrib and 1.505 J·m^−1^ including the midrib. Measurements for leaf physical strength traits from 10 to 20 leaves per species according to their size are suggested. To delve deeper, Onoda et al.^[5]^ partitioned each of three mechanical properties into three components: lamina thickness (*T*), tissue density (*ρ*), and toughness (or strength) per unit tissue density (*γ*) as the fundamental pillars underpinning these properties. To further examine these traits as predictors of litter mass loss, least-squares linear regression can be used to examine the relationships between species-specific traits and decomposition rates, as indicated by mass loss percent, decomposition constants (e.g., the *k* value), or the time required for 50% mass loss (represented by t_1/2_), along with stepwise multiple regression analyses to establish the optimal-fit model for predicting leaf litter decomposition processes. Plant species can be nested within different categories to illustrate the impact of species identity on the role of leaf physical strength traits as predictors of decomposition rates.

**Figure 2:**
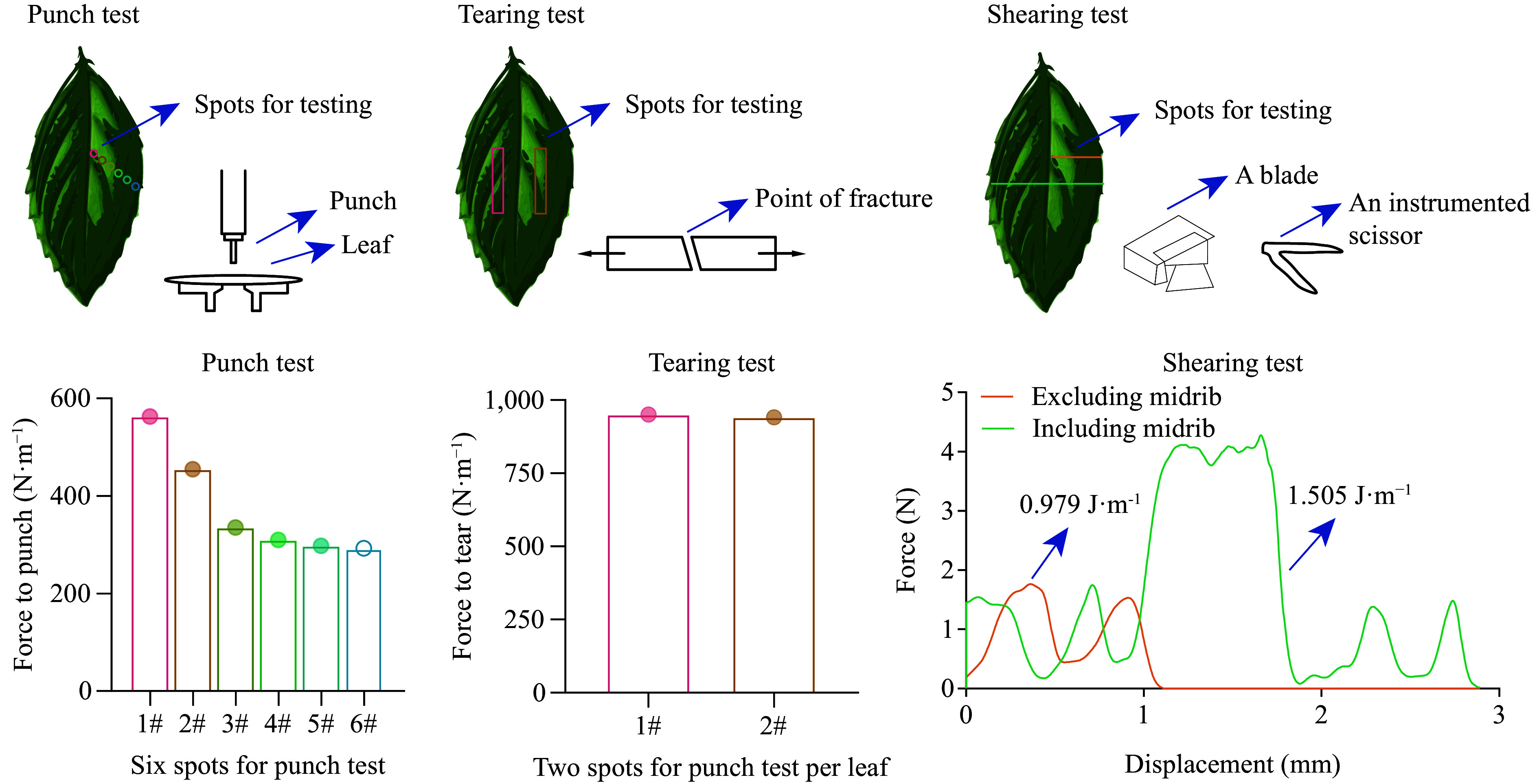
Illustration of common tests (inspired by Onoda et al.^[5]^) conducted to determine three leaf mechanical properties, namely, the punch test, tearing test and shearing test. The specific locations on the surfaces of fully matured fresh leaves where these tests are recommended to be performed are highlighted with colors that correspond to those found in the chart. These tests yield three mechanical properties: force to punch (*F*_*p*_, in units of N·m^−1^), force to tear (*F*_*t*_, N·m^−1^), and work to shear (*W*_*s*_, J·m^−1^). Using the leaves from *Eucalyptus globulus* as an example, the results from each of these tests are presented. Note that for needle-like or scale-like leaves, only tearing and shearing tests are applicable.

Dating back to 1994, Bergvinson et al.^[16]^ pioneered the development of an instron technique to measure *F*_*p*_ (force to punch) in maize (*Zea mays* L.) leaves and its relationship to the resistance of maize against larvae of *Ostrinia nubilalis*, a notorious pest known for feeding on plant tissues. Such leaf properties were therefore employed as a trait to assess plant resistance to herbivory within the field of plant-herbivory science. Additionally, other mechanical properties have found wide applications in the field of materials and food science, particularly in the assessment of food product texture^[17]^. The substantial interspecific variations observed in leaf mechanical properties at the global scale^[5]^ make them valuable indicators of diverse ecological processes in ecology. For leaf-litter decomposition, the mechanical resistance in leaf samples can exert significant legacy effects on the consequences of litter decomposition rates and subsequent nutrient cycling. However, seldom appreciated in the past, leaf mechanics are considered key factors in regulating leaf-litter decomposition (Fig. 1). The exploration of these properties as potential predictors of decomposition rates has been relatively limited. For example, one intriguing question that may pique people's curiosity is whether leaf mechanics can serve as crucial complements to the extensively studied leaf chemical traits in governing litter decomposition.

It is widely documented that plant carbon, nitrogen, lignin, and their ratios are consistently the most reliable indicators of litter decomposition rates. The C/N ratio reflects the proportion of carbohydrates to protein or the ratio of carbohydrate plus lignin to protein^[18]^. Notably, the C/N ratio describes the relative amounts of carbon and nitrogen, without revealing how these elements are distributed among the essential chemical components of the cell. Lignin plays a significant role in controlling decomposition rates by resisting enzymatic breakdown and physically impeding the decay of other chemical fractions (such as cellulose) within the leaf cell^[19]^. Analogously, plant physical strength also affects decomposition by exerting mechanical resistance against decomposers. Higher levels of leaf mechanical resistance are typically associated with reduced accessibility for leaf litter-colonized decomposers^[10]^. The potential enhancement of explanatory capacity in leaf-litter decomposition rates or litter mass loss through the incorporation of leaf mechanical resistance alongside leaf lignin contents is a topic open for discussion.

In regard to leaf structural characteristics, leaf mass per area (LMA) is a widely used generic trait in the field of leaf-litter decomposition. This trait, by definition, is a product of lamina thickness and leaf tissue density, and given that this metric is easy to measure, it has often served as a surrogate for leaf mechanical properties^[20]^. In fact, it has been used more frequently than leaf mechanical properties (e.g., *F*_*p*_, *F*_*t*_, and *W*_*s*_) in regard to leaf physical traits. The question is whether this trait can be used comprehensively in place of '*true'* leaf mechanical properties to reflect leaf litter resistance to decomposition. We suggest that relying solely on this surrogate overlooks the importance of variations in toughness (strength) per unit tissue density; this attribute may contribute significantly to the overall variation in leaf resistance against decomposers. In fact, according to reports by Onoda et al.^[5]^, tissue properties can play a more substantial role in determining resistance than lamina thickness and leaf tissue density, the two components of LMA. Onoda et al.^[5]^ partitioned the interspecific variations in *W*_*s*_ into different components. The results showed that lamina thickness contributed 26% of the total variance, leaf tissue density contributed 18%, and toughness per unit tissue density contributed 56%. Accordingly, LMA does not directly represent the actual mechanical properties, and the variation in toughness (strength) per unit tissue density, as proposed by Onoda et al., is not captured by this generic trait.

Many plant leaf functional traits are intertwined rather than isolated and exhibit associations and coordinated relationships that allow plants to adapt to changing external environments. For example, fibre and lignin content are likely major contributors to leaf toughness^[13]^. Leaf mechanical properties are also influenced by other plant traits, such as leaf material properties, thickness, age, and venation pattern, as well as by environmental conditions (e.g., sunlight or shade) and disturbance history (e.g., livestock browsing or forest fires)^[5]^. Although these traits are coordinated, according to Enrico et al.^[21]^, the precise values of one indicator cannot be fully reliably predicted solely based on the values of another, and it is also not advisable to 'fill in' missing data for certain trait indicators by predicting them solely based on another when available. Different traits should be examined individually or in an interplay manner. For leaf physical strength traits, fractures can occur either in tension or shear. Tension fractures typically occur at the weak zone near the intercalary meristem^[13]^, whereas shear work is experienced when forces act across the entire cross-sectional area of leaves, including the plant stems as a whole. Such shear work provides a comprehensive measure of the overall strength required to shear a leaf. As seen, even the three aforementioned mechanical properties (i.e., *F*_*p*_, *F*_*t*_, and *W*_*s*_) cannot be substituted for each other as potential predictors of the rates and patterns of leaf-litter decay. Nevertheless, to the best of our knowledge, no study has comprehensively examined all three mechanical properties in a single leaf-litter decomposition experiment. When considering the diverse plant functional traits associated with leaf-litter decomposition, it is important to recognize that these traits cannot be simply assumed to be interchangeable. In light of this, we propose a framework for categorizing these traits into three distinct groups, allowing for meaningful comparisons as potential predictors of leaf-litter decomposition processes (Fig. 3).

**Figure 3:**
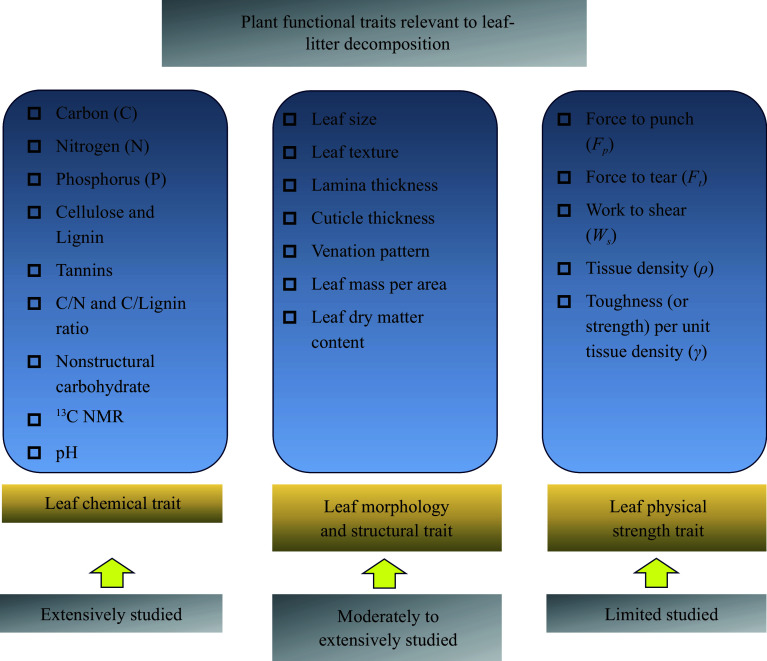
A framework summarizing the pivotal plant functional traits pertaining to leaf-litter decomposition. These traits are categorized into three groups: leaf chemical traits, leaf morphology and structural traits, and leaf physical strength traits. Among them, leaf physical strength traits have only received limited attention based on our literature survey.

Previously, a study conducted by Cornelissen & Thompson^[22]^ involving 48 Argentine species and 72 British species revealed a robust correlation between leaf toughness (tensile strength) and litter mass loss across all species. Compared to deciduous species, evergreens from these species typically exhibited inherently slower growth, characterized by lower specific leaf area and higher tensile strength, resulting in more resistant leaf litter against decomposition. Based on 38 British herbaceous species^[23]^, graminoid monocots were found to have physically tougher leaves than herbaceous dicots, and leaf tensile strength was clearly related to litter decomposition. By examining leaf samples from 52 angiosperms spanning a climatic gradient in central-western Argentina^[11]^, Pérez-Harguindeguy demonstrated the critical role of leaf tensile strength in connecting plant quality to decomposition patterns at the ecosystem level. This trait was recommended by authors due to its being easy and quick to measure across a diverse array of species. Another study focused on nine Mediterranean shrubs and trees in two distinct ecosystems, concluding that leaf toughness (measured as pressure required to penetrate the leaf blade) explained litter mass loss during both the leaching phase and the entire decomposition period^[24]^. Here, the ratio of toughness to phosphorus was also recognized as a crucial predictor of litter mass loss. These compelling findings provide solid evidence supporting the notion that leaf physical strength traits play a vital role in leaf-litter decomposition. However, it is important to note that studies investigating leaf mechanics within the context of leaf-litter decomposition experiments are still severely limited, leaving numerous questions unanswered. For instance, it remains unclear how leaf mechanics can enhance the predictive power of leaf decomposition and whether they can be effectively integrated into existing models to enhance our comprehension of this process. Specifically, it is important to investigate how these traits contribute uniquely to interspecific variations in decomposition processes in comparison to commonly measured plant chemical traits. Moreover, considering the irreplaceability of different traits, it is crucial to determine the individual or combined contributions of the aforementioned three mechanical properties in explaining litter decomposition. It is also essential to assess the generalizability of existing results when extrapolating predictors to a broader range of growth forms, species, habitats, and ecosystems. Particularly, when litter is subjected to different climatic zones, disturbance histories, or is mixed with leaf litter of other species, it is vital to determine if these traits can still reliably predict the decomposability ranking across species over time at both individual and community levels. For instance, wildfire often acts as a significant natural disturbance agent of vegetation and plays a role in contributing to the long-term carbon sink^[25]^. Taking wildfire as a typical case, we postulate that changes in plant traits, such as mechanical properties, within species that regenerate after wildfire disturbances could profoundly impact carbon balance by modulating litter decomposition processes. Furthermore, underlying explanations regarding the leaf mechanical resistance to decomposers that colonize the leaf litter are also needed.

As a final point, it is important to acknowledge that certain physical strength traits may lose some of their biological relevance in species with nonplanar photosynthetic organs. For instance, when dealing with needle-like or scale-like leaves, measuring the force required to puncture the leaf becomes challenging. Moreover, assessing physical strength traits is relatively cost-effective, nondestructive, and can be easily measured, although specialized equipment is often necessary.

## Closing remarks

Potential research gaps exist regarding the employment of leaf physical strength traits as candidate predictors of leaf-litter decomposability. Whether the focus is on the traits themselves or their evolutionary foundations, it is crucial to recognize that leaf chemical and physical traits are not mutually exclusive and, more importantly, cannot be substituted for one another. The significance of leaf physical strength traits in elucidating the ecological processes of leaf-litter decomposition is highlighted in this study, along with guidelines detailing precise locations on leaf surfaces and methods for measuring three commonly assessed leaf mechanical properties. Their connections and distinctions from other functional traits, such as LMA, a widely used physical trait, have been explained. Additionally, we elucidated the widely but improperly used term 'toughness' and introduced the more proper phrase 'leaf physical strength trait' to indicate leaf mechanical properties in this topic. Building upon these insights, we further presented a novel framework that classifies the pivotal plant functional traits related to leaf-litter decomposition into three major categories. Many unresolved inquiries regarding leaf physical strength traits as predictors of leaf-litter decomposability remain open for discussion and these traits deserve more attention compared to the extensively studied leaf chemical traits, which are currently the focus of research.

## SUPPLEMENTARY DATA

Supplementary data to this article can be found online.

## Data Availability

Data sharing is not applicable to this article as no datasets were generated or analyzed during the current study.

## References

[1] (2023). Forest understory vegetation study: current status and future trends. Forestry Research.

[2] (2016). Understanding the dominant controls on litter decomposition. Journal of Ecology.

[3] (2008). Plant species traits are the predominant control on litter decomposition rates within biomes worldwide. Ecology Letters.

[4] (2004). The worldwide leaf economics spectrum. Nature.

[5] (2011). Global patterns of leaf mechanical properties. Ecology Letters.

[6] (2012). A plant economics spectrum of litter decomposability. Functional Ecology.

[7] (2022). Temporal shifts in the explanatory power and relative importance of litter traits in regulating litter decomposition. Forest Ecosystems.

[8] (2017). Relationships among leaf functional traits, litter traits, and mass loss during early phases of leaf litter decomposition in 12 woody plant species. Oecologia.

[9] (2013). Effect of leaf litter characteristics on leaf conditioning and on consumption by *Gammarus pulex*. Freshwater Biology.

[10] (2016). Leaf litter decomposition in remote oceanic island streams is driven by microbes and depends on litter quality and environmental conditions. Freshwater Biology.

[11] (2000). Chemistry and toughness predict leaf litter decomposition rates over a wide spectrum of functional types and taxa in central Argentina. Plant and Soil.

[12] (2019). Soil fauna promote litter decomposition but do not alter the relationship between leaf economics spectrum and litter decomposability. Soil Biology and Biochemistry.

[13] (1995). A comparative study of the fracture properties of five grasses. Functional Ecology.

[b14] 14Atkins AG, Mai YW. 1985. Elastic and plastic fracture. Chichester, England: Ellis Horwood Ltd. 817 pp.

[15] (2013). New handbook for standardised measurement of plant functional traits worldwide. Australian Journal of Botany.

[16] (1995). Leaf profile of maize resistance factors to European corn borer, *Ostrinia nubilalis*. Journal of Chemical Ecology.

[17] (2021). Study on inhibition effects and mechanism of wheat starch retrogradation by polyols. Food Hydrocolloids.

[18] (1989). Nitrogen and lignin content as predictors of litter decay rates: a microcosm test. Ecology.

[19] (2020). Lignin lags, leads, or limits the decomposition of litter and soil organic carbon. Ecology.

[20] (2009). Causes and consequences of variation in leaf mass per area (LMA): a meta-analysis. New Phytologist.

[21] (2016). Leaf mechanical resistance in plant trait databases: comparing the results of two common measurement methods. Annals of Botany.

[22] (1999). Leaf structure and defence control litter decomposition rate across species and life forms in regional floras on two continents. New Phytologist.

[23] (1997). Functional leaf attributes predict litter decomposition rate in herbaceous plants. New Phytologist.

[24] (1993). Leaf decomposition in two Mediterranean ecosystems of southwest Spain: influence of substrate quality. Ecology.

[25] (2009). Fire in the earth system. Science.

