# Supplementary material for: Leaf mechanical properties as potential predictors of leaf-litter decomposability
Source: For Res (Fayettev). 2023 Sep 20;3:21. doi: 10.48130/FR-2023-0021 (PMC11524303; doi:10.48130/FR-2023-0021)
Supplement: Supplementary file 1 — Supplementary data to this article can be found online. [file FR-2023-0021-Suppl-TableS1.pdf]

Table S1 The list of selected publications (A total of 170 involved) that evaluate plant traits as predictors of litter decomposability between 2010—2022. Among them, those investigating the physical strength traits of leaves are highlighted in bold.

2010 (24 publications)

1. Alonso A, González-Muñoz N, Castro-Díez P. 2010. Comparison of leaf decomposition and macroinvertebrate colonization between exotic and native trees in a freshwater ecosystem. *Ecological Research* 25:647-53
2. Austin AT, Ballaré CL. 2010. Dual role of lignin in plant litter decomposition in terrestrial ecosystems. *Proceedings of the National Academy of Sciences* 107:4618-22
3. Baptist F, Yoccoz NG, Choler P. 2010. Direct and indirect control by snow cover over decomposition in alpine tundra along a snowmelt gradient. *Plant and Soil* 328:397-410
4. Bijayalaxmi Devi N, Yadava PS. 2010. Influence of climate and litter quality on litter decomposition and nutrient release in sub-tropical forest of Northeast India. *Journal of Forestry Research* 21:143-50
5. Bonanomi G, Incerti G, Antignani V, Capodilupo M, Mazzoleni S. 2010. Decomposition and nutrient dynamics in mixed litter of Mediterranean species. *Plant and Soil* 331:481-96
6. Cardelús CL. 2010. Litter decomposition within the canopy and forest floor of three tree species in a tropical lowland rain forest, Costa Rica. *Biotropica* 42:300-08
7. Castro H, Fortunel C, Freitas H. 2010. Effects of land abandonment on plant litter decomposition in a Montado system: Relation to litter chemistry and community functional parameters. *Plant and Soil* 333:181-90
8. Coq S, Souquet J-M, Meudec E, Cheynier V, Hättenschwiler S. 2010. Interspecific variation in leaf litter tannins drives decomposition in a tropical rain forest of French Guiana. *Ecology* 91:2080-91
9. Hättenschwiler S, Jørgensen HB. 2010. Carbon quality rather than stoichiometry controls litter decomposition in a tropical rain forest: Decomposition in a tropical rain forest. *Journal of Ecology* 98:754-63
10. Hobbie SE, Oleksyn J, Eissenstat DM, Reich PB. 2010. Fine root decomposition rates do not mirror those of leaf litter among temperate tree species. *Oecologia* 162:505-13
11. Jacob M, Viedenz K, Polle A, Thomas FM. 2010. Leaf litter decomposition in temperate deciduous forest stands with a decreasing fraction of beech (Fagus sylvatica). *Oecologia* 164:1083-94
12. Kurokawa H, Peltzer DA, Wardle DA. 2010. Plant traits, leaf palatability and litter decomposability for co-occurring woody species differing in invasion status and nitrogen fixation ability. *Functional Ecology* 24:513-23
13. Laganière J, Paré D, Bradley RL. 2010. How does a tree species influence litter decomposition? Separating the relative contribution of litter quality, litter mixing, and forest floor conditions. *Canadian Journal of Forest Research* 40:465-75
14. Lamers JPA, Martius C, Khamzina A, Matkarimova M, Djumaeva D, et al. 2010. Green

foliage decomposition in tree plantations on degraded, irrigated croplands in Uzbekistan, Central Asia. *Nutrient Cycling in Agroecosystems* 87:249-60

15. Laughlin DC, Leppert JJ, Moore MM, Sieg CH. 2010. A multi-trait test of the leaf-height-seed plant strategy scheme with 133 species from a pine forest flora. *Functional Ecology* 24:493-501
16. Liu P, Huang J, Sun OJ, Han X. 2010. Litter decomposition and nutrient release as affected by soil nitrogen availability and litter quality in a semiarid grassland ecosystem. *Oecologia* 162:771-80
17. Martínez G I, Zagal V E, Ovalle M C, Coûteaux M-M, Stolpe NB, et al. 2010. Litter decomposition of Acacia caven (Molina) Molina and Lolium multiflorum Lam. in mediterranean climate ecosystems. *Chilean journal of agricultural research* 70:454-64
18. Nikolaidou AE, Pavlatou-Ve AK, Kostopoulou SK, Mamolos AP, Kalburtji KL. 2010. Litter quality and decomposition of Vitis vinifera L. residues under organic and conventional farming systems. *European Journal of Soil Biology* 46:208-17
19. Nikula S, Vapaavuori E, Manninen S. 2010. Urbanization-related changes in European aspen (Populus tremula L.): Leaf traits and litter decomposition. *Environmental Pollution* 158:2132-42
20. Sánchez-Andrés R, Sánchez-Carrillo S, Alatorre LC, Cirujano S, Álvarez-Cobelas M. 2010. Litterfall dynamics and nutrient decomposition of arid mangroves in the Gulf of California: Their role sustaining ecosystem heterotrophy. *Estuarine, Coastal and Shelf Science* 89:191-99
21. Siegenthaler A, Buttler A, Bragazza L, Heijden EVD, Grosvernier P, et al. 2010. Litter-and ecosystem-driven decomposition under elevated CO2 and enhanced N deposition in a Sphagnum peatland. *Soil Biology and Biochemistry* 42:968-77
22. Van Geffen KG, Poorter L, Sass-Klaassen U, Van Logtestijn RSP, Cornelissen JHC. 2010. The trait contribution to wood decomposition rates of 15 Neotropical tree species. *Ecology* 91:3686-97
23. Xu G, Hu Y, Wang S, Zhang Z, Chang X, et al. 2010. Effects of litter quality and climate change along an elevation gradient on litter mass loss in an alpine meadow ecosystem on the Tibetan plateau. *Plant Ecology* 209:257-68
24. Zhang C, Trofymow JA, Jamieson RC, Meng F-R, Gordon R, et al. 2010. Litter decomposition and nitrogen mineralization from an annual to a monthly model. *Ecological Modelling* 221:1944-53

<u>2011</u> (9 publications)

25. **Bakker MA, Carreño-Rocabado G, Poorter L. 2011. Leaf economics traits predict litter decomposition of tropical plants and differ among land use types: Leaf economics traits and decomposition. *Functional Ecology* 25:473-83**
    "Force to punch" has been integrated into the construction of the leaf economics spectrum for predicting the litter decomposition of tropical plants.
26. Bottollier-Curtet M, Charcosset J-Y, Planty-Tabacchi A-M, Tabacchi E. 2011. Degradation of native and exotic riparian plant leaf litter in a floodplain pond: Leaf litter degradation of

invasive riparian plants. *Freshwater Biology* 56:1798-810

27. Group CW, Moore TR, Trofymow JA, Prescott CE, Titus BD. 2011. Nature and nurture in the dynamics of C, N and P during litter decomposition in Canadian forests. *Plant and Soil* 339:163-75

28. Li L-J, Zeng D-H, Yu Z-Y, Fan Z-P, Yang D, et al. 2011. Impact of litter quality and soil nutrient availability on leaf decomposition rate in a semi-arid grassland of Northeast China. *Journal of Arid Environments* 75:787-92

29. Nikula S, Manninen S, Vapaavuori E, Pulkkinen P. 2011. Growth, leaf traits and litter decomposition of roadside hybrid aspen (Populus tremula L.×P. tremuloides Michx.) clones. *Environmental Pollution* 159:1823-30

30. Pakeman RJ, Eastwood A, Scobie A. 2011. Leaf dry matter content as a predictor of grassland litter decomposition: A test of the 'mass ratio hypothesis'. *Plant and Soil* 342:49-57

31. Parsons SA, Lawler IR, Congdon RA, Williams SE. 2011. Rainforest litter quality and chemical controls on leaf decomposition with near-infrared spectrometry. *Journal of Plant Nutrition and Soil Science* 174:710-20

32. Silva LVB, Vasconcelos HL. 2011. Plant palatability to leaf-cutter ants (Atta laevigata) and litter decomposability in a Neotropical woodland savanna. *Austral Ecology* 36:504-10

33. Sundqvist MK, Giesler R, Wardle DA. 2011. Within-and across-species responses of plant traits and litter decomposition to elevation across contrasting vegetation types in subarctic tundra. *PLoS ONE* 6:e27056

2012 (14 publications)

34. Almagro M, Martínez-Mena M. 2012. Exploring short-term leaf-litter decomposition dynamics in a Mediterranean ecosystem: Dependence on litter type and site conditions. *Plant and Soil* 358:323-35

35. Aponte C, García LV, Marañón T. 2012. Tree species effect on litter decomposition and nutrient release in mediterranean oak forests changes over time. *Ecosystems* 15:1204-18

36. Arthur MA, Bray SR, Kuchle CR, McEwan RW. 2012. The influence of the invasive shrub, Lonicera maackii, on leaf decomposition and microbial community dynamics. *Plant Ecology* 213:1571-82

37. Bengtsson J, Janion C, Chown SL, Leinaas HP. 2012. Litter decomposition in fynbos vegetation, South Africa. *Soil Biology and Biochemistry* 47:100-05

38. **Danger M, Cornut J, Elger A, Chauvet E. 2012. Effects of burial on leaf litter quality, microbial conditioning and palatability to three shredder taxa: Leaf litter burial and palatability. *Freshwater Biology* 57:1017-30**

    Leaf litter palatability to shredders is suggested to be negatively related to toughness (force to punch).

39. Demessie A, Singh BR, Lal R, Strand LT. 2012. Leaf litter fall and litter decomposition under Eucalyptus and coniferous plantations in Gambo District, southern Ethiopia. *Acta Agriculturae Scandinavica, Section B - Soil & Plant Science* 62:467-76

40. Fernandes I, Pascoal C, Guimarães H, Pinto R, Sousa I, et al. 2012. Higher temperature

reduces the effects of litter quality on decomposition by aquatic fungi. *Freshwater Biology* 57:2306-17

41. **Ferreira V, Encalada AC, Graça MAS. 2012. Effects of litter diversity on decomposition and biological colonization of submerged litter in temperate and tropical streams. *Freshwater Science* 31:945-62**

"Force to punch" has been compared among 6 litter species from temperate deciduous forest and tropical rainforest, where toughness is suggested as a determinant litter characateristic for invertebtrate feeding and subsequently decomposition rate.

42. Lan Y, Cui B, You Z, Li X, Han Z, et al. 2012. Litter decomposition of six macrophytes in a eutrophic shallow lake (Baiyangdian Lake, China). *CLEAN - Soil, Air, Water* 40:1159-66

43. **Makkonen M, Berg MP, Handa IT, Hättenschwiler S, Van Ruijven J, et al. 2012. Highly consistent effects of plant litter identity and functional traits on decomposition across a latitudinal gradient. *Ecology Letters* 15:1033-41**

Toughness and tensile strength have been integrated into multiple linear regression model for predicting decomposition constant (e.g., *k*-value) across a latitudinal gradient.

44. Mooshammer M, Wanek W, Schnecker J, Wild B, Leitner S, et al. 2012. Stoichiometric controls of nitrogen and phosphorus cycling in decomposing beech leaf litter. *Ecology* 93:770-82

45. Waring BG. 2012. A meta-analysis of climatic and chemical controls on leaf litter decay rates in tropical forests. *Ecosystems* 15:999-1009

46. Wickings K, Grandy AS, Reed SC, Cleveland CC. 2012. The origin of litter chemical complexity during decomposition. *Ecology Letters* 15:1180-88

47. Yang X, Yang Z, Warren MW, Chen J. 2012. Mechanical fragmentation enhances the contribution of Collembola to leaf litter decomposition. *European Journal of Soil Biology* 53:23-31

2013 (8 publications)

48. **Casas JJ, Larrañaga A, Menéndez M, Pozo J, Basaguren A, et al. 2013. Leaf litter decomposition of native and introduced tree species of contrasting quality in headwater streams: How does the regional setting matter? *Science of the Total Environment* 458-460:197-208**

Leaf toughness after leaching has been compared between native and introduced tree species and linked with the breakdown rates of leaf litter originating from contrasting climates.

49. **Foucreau N, Puijalon S, Hervant F, Piscart C. 2013. Effect of leaf litter characteristics on leaf conditioning and on consumption by Gammarus pulex. *Freshwater Biology* 58:1672-81**

The time ($t_{1/2}$) needed for a decrease by 50% of the initial value of the toughness (indicated by "Work to punch") or specific toughness has been determined and leaf consumption rate by Gammarus pulex is suggested to be negatively correated with the specific toughness.

50. García-Palacios P, Maestre FT, Kattge J, Wall DH. 2013. Climate and litter quality differently modulate the effects of soil fauna on litter decomposition across biomes. *Ecology Letters* 16:1045-53

51. Jackson BG, Peltzer DA, Wardle DA. 2013. Are functional traits and litter decomposability coordinated across leaves, twigs and wood? A test using temperate rainforest tree species. *Oikos* 122:1131-42

52. Jackson BG, Peltzer DA, Wardle DA. 2013. The within-species leaf economic spectrum does not predict leaf litter decomposability at either the within-species or whole community levels. *Journal of Ecology* 101:1409-19

53. **Rahman MM, Tsukamoto J. 2013. Leaf traits, litter decomposability and forest floor dynamics in an evergreen-and a deciduous-broadleaved forest in warm temperate Japan. *Forestry* 86:441-51**

Leaf toughness is suggested to be closely related to functional type among different species. Particularly, high leaf toughness of evergreens renders their litter more resistant to moistening than the litter of the deciduous species, thus negatively impacting litter decay.

54. **Rahman MM, Tsukamoto J, Tokumoto Y, Shuvo MAR. 2013. The role of quantitative traits of leaf litter on decomposition and nutrient cycling of the forest ecosystems. *Journal of Forest and Environmental Science* 29:38-48**

The influence of leaf toughness on decomposition rate in forest ecosystem has been examined, comparing with leaf mass per area and chemical properties (mainly nitrogen and lignin).

55. Zhu J, Yang W, He X. 2013. Temporal dynamics of abiotic and biotic factors on leaf litter of three plant species in relation to decomposition rate along a subalpine elevation gradient. *PLoS ONE* 8:e62073

2014 (13 publications)

56. Alexander HD, Arthur MA. 2014. Increasing red maple leaf litter alters decomposition rates and nitrogen cycling in historically oak-dominated forests of the eastern US. *Ecosystems* 17:1371-83

57. Berg B. 2014. Decomposition patterns for foliar litter – A theory for influencing factors. *Soil Biology and Biochemistry* 78:222-32

58. Cornwell WK, Weedon JT. 2014. Decomposition trajectories of diverse litter types: A model selection analysis. *Methods in Ecology and Evolution* 5:173-82

59. Hangs RD, Schoenau JJ, Van Rees KCJ, Bélanger N, Volk T. 2014. Leaf litter decomposition and nutrient-release characteristics of several willow varieties within short-rotation coppice plantations in Saskatchewan, Canada. *BioEnergy Research* 7:1074-90

60. Hristovski S, Berg B, Melovski L. 2014. Limitless decomposition in leaf litter of Common beech: Patterns, nutrients' and heavy metal's dynamics. *Pedobiologia* 57:131-38

61. Jiang X, Cao L, Zhang R, Yan L, Mao Y, et al. 2014. Effects of nitrogen addition and litter properties on litter decomposition and enzyme activities of individual fungi. *Applied Soil*

Ecology 80:108-15

62. Li S, Liu W-Y, Li D-W, Li Z-X, Song L, et al. 2014. Slower rates of litter decomposition of dominant epiphytes in the canopy than on the forest floor in a subtropical montane forest, southwest China. *Soil Biology and Biochemistry* 70:211-20
63. Mincheva T, Barni E, Varese GC, Brusa G, Cerabolini B, et al. 2014. Litter quality, decomposition rates and saprotrophic mycoflora in Fallopia japonica (Houtt.) Ronse Decraene and in adjacent native grassland vegetation. *Acta Oecologica* 54:29-35
64. **Pan X, Cornelissen JHC, Zhao WW, Liu GF, Hu YK, et al. 2014. Experimental evidence that the Ornstein-Uhlenbeck model best describes the evolution of leaf litter decomposability. *Ecology and Evolution* 4:3339-49**

    Leaf tensile strengh as plant traits has been incorporated into Ornstein-Uhlenbeck model, which best describes the evolution of leaf litter decomposability.
65. Purahong W, Kapturska D, Pecyna MJ, Schulz E, Schloter M, et al. 2014. Influence of different forest system management practices on leaf litter decomposition rates, nutrient dynamics and the activity of ligninolytic enzymes: A case study from Central European forests. *PLoS ONE* 9:e93700
66. Tu L-H, Hu H-L, Hu T-X, Zhang J, Li X-W, et al. 2014. Litterfall, litter decomposition, and nutrient dynamics in two subtropical bamboo plantations of China. *Pedosphere* 24:84-97
67. Walela C, Daniel H, Wilson B, Lockwood P, Cowie A, et al. 2014. The initial lignin: Nitrogen ratio of litter from above and below ground sources strongly and negatively influenced decay rates of slowly decomposing litter carbon pools. *Soil Biology and Biochemistry* 77:268-75
68. Zhang X, Song C, Mao R, Yang G, Tao B, et al. 2014. Litter mass loss and nutrient dynamics of four emergent macrophytes during aerial decomposition in freshwater marshes of the Sanjiang plain, Northeast China. *Plant and Soil* 385:139-47

2015 (14 publications)

69. Berg B, Kjønaas OJ, Johansson MB, Erhagen B, Åkerblom S. 2015. Late stage pine litter decomposition: Relationship to litter N, Mn, and acid unhydrolyzable residue (AUR) concentrations and climatic factors. *Forest Ecology and Management* 358:41-47
70. Castellano MJ, Mueller KE, Olk DC, Sawyer JE, Six J. 2015. Integrating plant litter quality, soil organic matter stabilization, and the carbon saturation concept. *Global Change Biology* 21:3200-09
71. Dale SE, Turner BL, Bardgett RD. 2015. Isolating the effects of precipitation, soil conditions, and litter quality on leaf litter decomposition in lowland tropical forests. *Plant and Soil* 394:225-38
72. **Eichenberg D, Trogisch S, Huang Y, He J-S, Bruelheide H. 2015. Shifts in community leaf functional traits are related to litter decomposition along a secondary forest succession series in subtropical China. *Journal of Plant Ecology* 8:401-10**

    Leaf toughness has been considered as a form of physical resistance, a type of trait function; shifts in this functional trait have been connected to litter decomposition along a secondary forest succession series in subtropical China.

73. Frainer A, Moretti MS, Xu W, Gessner MO. 2015. No evidence for leaf-trait dissimilarity effects on litter decomposition, fungal decomposers, and nutrient dynamics. *Ecology* 96:550-61

74. Keiluweit M, Nico P, Harmon ME, Mao J, Pett-Ridge J, et al. 2015. Long-term litter decomposition controlled by manganese redox cycling. *Proceedings of the National Academy of Sciences* 112:E5253-E60

75. Letts B, Lamb EG, Mischkolz JM, Romo JT. 2015. Litter accumulation drives grassland plant community composition and functional diversity via leaf traits. *Plant Ecology* 216:357-70

Increasing litter mass can cause rapid declines in plant diversity and a convergence in trait structure toward dominance by taller graminoids with increased density of leaf tissues.

76. Pan X, Berg MP, Butenschoen O, Murray PJ, Bartish IV, et al. 2015. Larger phylogenetic distances in litter mixtures: Lower microbial biomass and higher C/N ratios but equal mass loss. *Proceedings of the Royal Society B: Biological Sciences* 282:20150103

Toughness has been considered as one of phylogenetic signal traits to explore how different traits influence litter mass loss from an evolutionary perspective.

77. Paudel E, Dossa GGO, De Blécourt M, Beckschäfer P, Xu J, et al. 2015. Quantifying the factors affecting leaf litter decomposition across a tropical forest disturbance gradient. *Ecosphere* 6:1-20

78. Pérez Harguindeguy N, Cortez J, Garnier E, Gillon D, Poca M. 2015. Predicting leaf litter decomposability: An exploratory assessment of leaf traits, litter traits and spectral properties in six Mediterranean herbaceous species. *Ecología Austral* 25:54-64

79. Riggs CE, Hobbie SE, Cavender-Bares J, Savage JA, Wei X. 2015. Contrasting effects of plant species traits and moisture on the decomposition of multiple litter fractions. *Oecologia* 179:573-84

80. Wang J, You Y, Tang Z, Liu S, Sun OJ. 2015. Variations in leaf litter decomposition across contrasting forest stands and controlling factors at local scale. *Journal of Plant Ecology* 8:261-72

81. Wang Y, Gong J-R, Liu M, Luo Q, Xu S, et al. 2015. Effects of land use and precipitation on above-and below-ground litter decomposition in a semi-arid temperate steppe in Inner Mongolia, China. *Applied Soil Ecology* 96:183-91

82. Zhang X, Wang W. 2015. Control of climate and litter quality on leaf litter decomposition in different climatic zones. *Journal of Plant Research* 128:791-802

2016 (18 publications)

83. Barba J, Lloret F, Yuste JC. 2016. Effects of drought-induced forest die-off on litter decomposition. *Plant and Soil* 402:91-101

84. Bosco T, Bertiller MB, Carrera AL. 2016. Combined effects of litter features, UV radiation, and soil water on litter decomposition in denuded areas of the arid Patagonian Monte. *Plant and Soil* 406:71-82

85. Boyero L, Pearson RG, Hui C, Gessner MO, Pérez J, et al. 2016. Biotic and abiotic variables influencing plant litter breakdown in streams: A global study. *Proceedings of the Royal Society B: Biological Sciences* 283:20152664

86. Bradford MA, Berg B, Maynard DS, Wieder WR, Wood SA. 2016. Understanding the dominant controls on litter decomposition. *Journal of Ecology* 104:229-38

87. Ferreira V, Raposeiro PM, Pereira A, Cruz AM, Costa AC, et al. 2016. Leaf litter decomposition in remote oceanic island streams is driven by microbes and depends on litter quality and environmental conditions. *Freshwater Biology* 61:783-99

"Force to punch" has been compared among different species, proving significant in influencing microbial colonization and decomposition rates of leaf litter within both coarse and fine mesh bags.

88. Gao J, Kang F, Han H. 2016. Effect of litter quality on leaf-litter decomposition in the context of home-field advantage and non-additive effects in temperate forests in China. *Polish Journal of Environmental Studies* 25:1911-20

89. García-Palacios P, Prieto I, Ourcival J-M, Hättenschwiler S. 2016. Disentangling the litter quality and soil microbial contribution to leaf and fine root litter decomposition responses to reduced rainfall. *Ecosystems* 19:490-503

90. García-Palacios P, Shaw EA, Wall DH, Hättenschwiler S. 2016. Temporal dynamics of biotic and abiotic drivers of litter decomposition. *Ecology Letters* 19:554-63

91. García-Palacios P, McKie BG, Handa IT, Frainer A, Hättenschwiler S. 2016. The importance of litter traits and decomposers for litter decomposition: A comparison of aquatic and terrestrial ecosystems within and across biomes. *Functional Ecology* 30:819-29

Toughness is regarded as a pivotal trait for predicting litter decomposition in a comparison test across aquatic and terrestrial ecosystems, both within and across different biomes.

92. Li H, Liu Y, Li J, Zhou X, Li B. 2016. Dynamics of litter decomposition of dieback Phragmites in Spartina-invaded salt marshes. *Ecological Engineering* 90:459-65

93. Lovett GM, Arthur MA, Crowley KF. 2016. Effects of calcium on the rate and extent of litter decomposition in a northern hardwood forest. *Ecosystems* 19:87-97

94. Mao B, Mao R, Hu Y, Huang Y, Zeng D. 2016. Decomposition of Mongolian pine litter in the presence of understory species in semi-arid northeast China. *Journal of Forestry Research* 27:329-37

95. Portillo-Estrada M, Pihlatie M, Korhonen JFJ, Levula J, Frumau AKF, et al. 2016. Climatic controls on leaf litter decomposition across European forests and grasslands revealed by reciprocal litter transplantation experiments. *Biogeosciences* 13:1621-33

96. Wang J, You Y, Tang Z, Sun X, Sun OJ. 2016. A comparison of decomposition dynamics among green tree leaves, partially decomposed tree leaf litter and their mixture in a warm temperate forest ecosystem. *Journal of Forestry Research* 27:1037-45

97. Xu Z, Zhu J, Wu F, Liu Y, Tan B, et al. 2016. Effects of litter quality and climate change along an elevational gradient on litter decomposition of subalpine forests, Eastern Tibetan Plateau, China. *Journal of Forestry Research* 27:505-11

98. Yue K, Peng C, Yang W, Peng Y, Zhang C, et al. 2016. Degradation of lignin and cellulose during foliar litter decomposition in an alpine forest river. *Ecosphere* 7:e01523

99. Yue K, Yang W, Peng C, Peng Y, Zhang C, et al. 2016. Foliar litter decomposition in an alpine forest meta-ecosystem on the eastern Tibetan Plateau. *Science of the Total Environment* 566-567:279-87

100. Zhu W, Wang J, Zhang Z, Ren F, Chen L, et al. 2016. Changes in litter quality induced by nutrient addition alter litter decomposition in an alpine meadow on the Qinghai-Tibet Plateau. *Scientific Reports* 6:34290

2017 (6 publications)

101. Barbe L, Jung V, Prinzing A, Bittebiere AK, Butenschoen O, et al. 2017. Functionally dissimilar neighbors accelerate litter decomposition in two grass species. *New Phytologist* 214:1092-102

"Force to punch" has been taken into consideration when examining the impacts of functional dissimilarity to neighborhood on functional traits of focal plants and when evaluating the effects of functional traits on litter mass loss.

102. Krishna MP, Mohan M. 2017. Litter decomposition in forest ecosystems: A review. *Energy, Ecology and Environment* 2:236-49

103. Li S, Tong Y, Wang Z. 2017. Species and genetic diversity affect leaf litter decomposition in subtropical broadleaved forest in southern China. *Journal of Plant Ecology* 10:232-41

104. Liu G, Sun J, Tian K, Xiao D, Yuan X. 2017. Long-term responses of leaf litter decomposition to temperature, litter quality and litter mixing in plateau wetlands. *Freshwater Biology* 62:178-90

105. Szefer P, Carmona CP, Chmel K, Konečná M, Libra M, et al. 2017. Determinants of litter decomposition rates in a tropical forest: Functional traits, phylogeny and ecological succession. *Oikos* 126:1101-11

106. Zukswert JM, Prescott CE. 2017. Relationships among leaf functional traits, litter traits, and mass loss during early phases of leaf litter decomposition in 12 woody plant species. *Oecologia* 185:305-16

"Force to punch" is proposed as a significant trait in predicting litter decomposition among 12 woody plant species encompassing both phase I and phase II decomposing stages.

2018 (7 publications)

107. Djukic I, Kepfer-Rojas S, Schmidt IK, Larsen KS, Beier C, et al. 2018. Early stage litter decomposition across biomes. *Science of the Total Environment* 628-629:1369-94

108. Duan H, Wang L, Zhang Y, Fu X, Tsang Y, et al. 2018. Variable decomposition of two plant litters and their effects on the carbon sequestration ability of wetland soil in the Yangtze River estuary. *Geoderma* 319:230-38

109. Liu G, Wang L, Jiang L, Pan X, Huang Z, et al. 2018. Specific leaf area predicts dryland litter decomposition via two mechanisms. *Journal of Ecology* 106:218-29
110. Remy E, Wuyts K, Van Nevel L, De Smedt P, Boeckx P, et al. 2018. Driving factors behind litter decomposition and nutrient release at temperate forest edges. *Ecosystems* 21:755-71
111. Sánchez-Silva S, De Jong BHJ, Aryal DR, Huerta-Lwanga E, Mendoza-Vega J. 2018. Trends in leaf traits, litter dynamics and associated nutrient cycling along a secondary successional chronosequence of semi-evergreen tropical forest in South-Eastern Mexico. *Journal of Tropical Ecology* 34:364-77
112. Sun T, Hobbie SE, Berg B, Zhang H, Wang Q, et al. 2018. Contrasting dynamics and trait controls in first-order root compared with leaf litter decomposition. *Proceedings of the National Academy of Sciences* 115:10392-97
113. Wang Q, Kwak J-H, Choi W-J, Chang SX. 2018. Decomposition of trembling aspen leaf litter under long-term nitrogen and sulfur deposition: Effects of litter chemistry and forest floor microbial properties. *Forest Ecology and Management* 412:53-61

<u>2019</u> (5 publications)

114. De La Riva EG, Prieto I, Villar R. 2019. The leaf economic spectrum drives leaf litter decomposition in Mediterranean forests. *Plant and Soil* 435:353-66
115. Lin D, Wang F, Fanin N, Pang M, Dou P, et al. 2019. Soil fauna promote litter decomposition but do not alter the relationship between leaf economics spectrum and litter decomposability. *Soil Biology and Biochemistry* 136:107519
Structure-related trait such as toughness is suggested to be strongly related to decomposition rate. This trait has been integrated into the leaf economics spectrum (LES), and meso- and macrofauna presence does not significantly alter the relationships between LES and litter mass loss.
116. Pei G, Liu J, Peng B, Gao D, Wang C, et al. 2019. Nitrogen, lignin, C/N as important regulators of gross nitrogen release and immobilization during litter decomposition in a temperate forest ecosystem. *Forest Ecology and Management* 440:61-69
117. Vivanco L, Austin AT. 2019. The importance of macro-and micro-nutrients over climate for leaf litter decomposition and nutrient release in Patagonian temperate forests. *Forest Ecology and Management* 441:144-54
118. Zhang M, Cheng X, Geng Q, Shi Z, Luo Y, et al. 2019. Leaf litter traits predominantly control litter decomposition in streams worldwide. *Global Ecology and Biogeography* 28:1469-86
Toughness has been incorporated into the model in explaining litter decomposition rate in streams worldwide.

<u>2020</u> (10 publications)

119. Bryanin S, Kondratova A, Abramova E. 2020. Litter decomposition and nutrient dynamics in fire-affected larch forests in the Russian far east. *Forests* 11:882

120. Elias DMO, Robinson S, Both S, Goodall T, Majalap-Lee N, et al. 2020. Soil microbial community and litter quality controls on decomposition across a tropical forest disturbance gradient. *Frontiers in Forests and Global Change* 3:81

121. Esquivel J, Park BB, Casanoves F, Delgado D, Park GE, et al. 2020. Altitude and species identity drive leaf litter decomposition rates of ten species on a 2950 m altitudinal gradient in Neotropical rain forests. *Biotropica* 52:11-21

The leaf economic spectrum traits (SLA, N, P) and other traits related to leaf structure, longevity, and toughness (LDMC, F_t) play a role in predicting the decomposition rates of leaf litter.

122. Helsen K, Van Cleemput E, Bassi L, Somers B, Honnay O. 2020. Optical traits perform equally well as directly-measured functional traits in explaining the impact of an invasive plant on litter decomposition. *Journal of Ecology* 108:2000-11

123. Li Q, Zhang M, Geng Q, Jin C, Zhu J, et al. 2020. The roles of initial litter traits in regulating litter decomposition: A “common plot” experiment in a subtropical evergreen broadleaf forest. *Plant and Soil* 452:207-16

124. Mun S, Lee EJ. 2020. Litter decomposition rate and nutrient dynamics of giant ragweed (Ambrosia trifida L.) in the non-native habitat of South Korea. *Plant and Soil* 449:373-88

125. Park H-J, Lim S-S, Yang HI, Lee K-S, Park S-I, et al. 2020. Co-elevated CO2 and temperature and changed water availability do not change litter quantity and quality of pine and oak. *Agricultural and Forest Meteorology* 280:107795

126. Rosenfield MV, Keller JK, Clausen C, Cyphers K, Funk JL. 2020. Leaf traits can be used to predict rates of litter decomposition. *Oikos* 129:1589-96

127. Vinh TV, Allenbach M, Linh KTV, Marchand C. 2020. Changes in leaf litter quality during its decomposition in a tropical planted mangrove forest (Can Gio, Vietnam). *Frontiers in Environmental Science* 8:10

128. Zeng A, Hu W, Zeng C, Sun Z, Gao D. 2020. Litter decomposition and nutrient dynamics of native species (Cyperus malaccensis) and alien invasive species (Spartina alterniflora) in a typical subtropical estuary (Min River) in China. *Estuaries and Coasts* 43:1873-83

2021 (27 publications)

129. Ahmad R, Khuroo AA, Hamid M, Rashid I, Rather ZA. 2021. Disentangling the determinants of litter decomposition among invaded and uninvaded habitats: A field experiment from the Kashmir Himalaya. *Acta Oecologica* 110:103708

130. Akinyele AO, Donald-Amaeshi U. 2021. Leaf litter decomposition and nutrient release of three selected agroforestry tree species. *Agroforestry Systems* 95:559-70

131. Berg B, Sun T, Johansson M-B, Sanborn P, Ni X, et al. 2021. Magnesium dynamics in decomposing foliar litter - A synthesis. *Geoderma* 382:114756

132. Bonanomi G, Idbella M, Zotti M, Santorufo L, Motti R, et al. 2021. Decomposition and temperature sensitivity of fine root and leaf litter of 43 Mediterranean species. *Plant and Soil*

464:453-65

133. Cai A, Liang G, Yang W, Zhu J, Han T, et al. 2021. Patterns and driving factors of litter decomposition across Chinese terrestrial ecosystems. *Journal of Cleaner Production* 278:123964

134. Canessa R, Brink L, Saldaña A, Rios RS, Hättenschwiler S, et al. 2021. Relative effects of climate and litter traits on decomposition change with time, climate and trait variability. *Journal of Ecology* 109:447-58

Force to punch is suggested to have a negative correlation with decomposition rates, and trait effects on litter decomposition can gain in importance when including a greater variation in trait values.

135. De Marco A, Spaccini R, Virzo De Santo A. 2021. Differences in nutrients, organic components and decomposition pattern of Phillyrea angustifolia leaf litter across a low maquis. *Plant and Soil* 464:559-78

136. Fang X-M, Zhang X-L, Zong Y-Y, Li W-Q, Li J-J, et al. 2021. Responses of leaf litter decomposability to nitrogen and phosphorus additions are associated with cell wall carbohydrate composition in a subtropical plantation. *Plant and Soil* 467:359-72

137. Firmino VC, Brasil LS, Martins RT, Ligeiro R, Tonin A, et al. 2021. Litter decomposition of exotic and native plant species of agricultural importance in Amazonian streams. *Limnology* 22:289-97

138. Fu YM, Zhang XY, Qi DD, Feng FJ. 2021. Changes in leaf litter decomposition of primary Korean pine forests after degradation succession into secondary broad-leaved forests. *Ecology and Evolution* 11:12335-48

139. Guo L, Deng M, Yang S, Liu W, Wang X, et al. 2021. The coordination between leaf and fine root litter decomposition and the difference in their controlling factors. *Global Ecology and Biogeography* 30:2286-96

140. Hebert TA, Halvorson HM, Kuehn KA. 2021. A literature synthesis resolves litter intrinsic constraints on fungal dynamics and decomposition across standing dead macrophytes. *Oikos* 130:958-68

141. Hong J, Lu X, Ma X, Wang X. 2021. Five-year study on the effects of warming and plant litter quality on litter decomposition rate in a Tibetan alpine grassland. *Science of the Total Environment* 750:142306

142. Keiser AD, Warren R, Filley T, Bradford MA. 2021. Signatures of an abiotic decomposition pathway in temperate forest leaf litter. *Biogeochemistry* 153:177-90

143. Li R, Zhang Y, Yu D, Wang Y, Zhao X, et al. 2021. The decomposition of green leaf litter is less temperature sensitive than that of senescent leaf litter: An incubation study. *Geoderma* 381:114691

144. López-Rojo N, Pérez J, Pozo J, Basaguren A, Apodaka-Etxebarria U, et al. 2021. Shifts in key leaf litter traits can predict effects of plant diversity loss on decomposition in streams. *Ecosystems* 24:185-96

145. Martínez-García LB, Korthals GW, Brussaard L, Mainardi G, De Deyn GB. 2021. Litter quality drives nitrogen release, and agricultural management (organic vs. conventional) drives carbon loss during litter decomposition in agro-ecosystems. *Soil Biology and Biochemistry* 153:108115

146. Pastorelli R, Costagli V, Forte C, Viti C, Rompato B, et al. 2021. Litter decomposition: Little

evidence of the "home-field advantage" in a mountain forest in Italy. *Soil Biology and Biochemistry* 159:108300

147. Ramos SM, Graça MAS, Ferreira V. 2021. A comparison of decomposition rates and biological colonization of leaf litter from tropical and temperate origins. *Aquatic Ecology* 55:925-40

148. Rawlik K, Nowiński M, Jagodziński AM. 2021. Short life-fast death: Decomposition rates of woody plants leaf-and herb-litter. *Annals of Forest Science* 78:6

149. Severino Da Silva L, Sollenberger LE, Moura Kohmann M, Dubeux JC, Aryal P, et al. 2021. Litter mass and nitrogen disappearance in year-round nitrogen-fertilized grass and legume-grass forage systems. *Agronomy Journal* 113:5170-82

150. Seyfried GS, Dalling JW, Yang WH. 2021. Mycorrhizal type effects on leaf litter decomposition depend on litter quality and environmental context. *Biogeochemistry* 155:21-38

151. Tong R, Zhou B, Jiang L, Ge X, Cao Y, et al. 2021. Leaf litter carbon, nitrogen and phosphorus stoichiometry of Chinese fir (Cunninghamia lanceolata) across China. *Global Ecology and Conservation* 27:e01542

152. Wang W, Hu K, Huang K, Tao J. 2021. Mechanical fragmentation of leaf litter by fine root growth contributes greatly to the early decomposition of leaf litter. *Global Ecology and Conservation* 26:e01456

153. Yang J, Zhang Y, Fu C, Liang Z, Yue K, et al. 2021. Seasonal dynamics of organic components in fresh foliar litters at different gap positions in an alpine forest on the eastern Tibetan Plateau. *Journal of Soils and Sediments* 21:810-20

154. Yue K, Ni X, Fornara DA, Peng Y, Liao S, et al. 2021. Dynamics of calcium, magnesium, and manganese during litter decomposition in alpine forest aquatic and terrestrial ecosystems. *Ecosystems* 24:516-29

155. Zhang Y, Pennings SC, Liu Z, Li B, Wu J. 2021. Consistent pattern of higher lability of leaves from high latitudes for both native Phragmites australis and exotic Spartina alterniflora. *Functional Ecology* 35:2084-93

The decomposition rate of litter is negatively correlated with leaf toughness, tannin concentration, and lignin concentration, while positively correlated with calcium and magnesium concentrations.

2022 (15 publications)

156. Berg B, Lönn M. 2022. Long-term effects of climate and litter chemistry on rates and stable fractions of decomposing Scots pine and Norway spruce needle litter — A synthesis. *Forests* 13:125

157. Carrasco-Barea L, Llorens L, Romaní AM, Gispert M, Verdaguer D. 2022. Litter decomposition of three halophytes in a Mediterranean salt marsh: Relevance of litter quality, microbial activity and microhabitat. *Science of the Total Environment* 838:155743

158. Cheng C, Liu Z, Zhang Y, He Q, Li B, et al. 2022. Leaf litter decomposition and its drivers differ between an invasive and a native plant: Management implications. *Ecological*

Applications 32:e2739

159. Ganault P, Barantal S, Coq S, Hättenschwiler S, Lucas S, et al. 2022. Leaf litter morphological traits, invertebrate body mass and phylogenetic affiliation explain the feeding and feces properties of saprophagous macroarthropods. *European Journal of Soil Biology* 109:103383

160. Ge J, Ma B, Xu W, Zhao C, Xie Z. 2022. Temporal shifts in the relative importance of climate and leaf litter traits in driving litter decomposition dynamics in a Chinese transitional mixed forest. *Plant and Soil* 477:679-92

161. Hu X, Arif M, Ding D, Li J, He X, et al. 2022. Invasive plants and species richness impact litter decomposition in riparian zones. *Frontiers in Plant Science* 13:955656

162. Marler TE, Cruz GN. 2022. Temporal variation of litterfall and nutrient return of Serianthes nelsonii Merr. in a tropical karst forest. *Plants* 11:2310

163. Peng Y, Holmstrup M, Kappel Schmidt I, Ruggiero Bachega L, Schelfhout S, et al. 2022. Tree species identity is the predominant modulator of the effects of soil fauna on leaf litter decomposition. *Forest Ecology and Management* 520:120396

164. Rawlik K, Kasprowicz M, Nowiński M, Jagodziński AM. 2022. The afterlife of herbaceous plant species: A litter decomposition experiment in a temperate oak-hornbeam forest. *Forest Ecology and Management* 507:120008

165. Roeder M, Dossa GGO, Cornelissen JHC, Yang X, Nakamura A, et al. 2022. Liana litter decomposes faster than tree litter in a multispecies and multisite experiment. *Journal of Ecology* 110:2433-47

Lianas have lower leaf dry matter content and toughness than trees, resulting in their faster decomposition rate.

166. Su Z, Zhu X, Wang Y, Mao S, Shangguan Z. 2022. Litter C and N losses at different decomposition stages of Robinia pseudoacacia: The weaker effects of soil enzyme activities compared with those of litter quality and the soil environment. *Frontiers in Environmental Science* 10:956309

167. Sun Z, Tian P, Zhao X, Wang Y, Wang S, et al. 2022. Temporal shifts in the explanatory power and relative importance of litter traits in regulating litter decomposition. *Forest Ecosystems* 9:100072

Litter decomposition half-life (Weibull $t_{1/2}$) depends on factors like P, Ca, Mg, K, Na, condensed tannins, water saturation capacity, and tensile strength. On the other hand, litter mean residence time (Weibull MRT) is primarily affected by factors such as P, Ca, condensed tannins, lignin, non-structural carbohydrates (NSC), and tensile strength.

168. Wang H, Xu X, Wang Z, Cao R, Zheng B, et al. 2022. Abnormal litter induced by typhoon disturbances had higher rates of mass loss and carbon release than physiological litter in coastal subtropical urban forest ecosystems. *Forests* 13:1819

169. Zan P, Mao Z, Sun T. 2022. Effects of soil fauna on litter decomposition in Chinese forests: A meta-analysis. *Peerj* 10:e12747

170. Zhao Y-Y, Li Z-T, Xu T, Lou A-r. 2022. Leaf litter decomposition characteristics and controlling factors across two contrasting forest types. *Journal of Plant Ecology* 15:1285-301
